# *In situ* observations of coral bleaching in the central Saudi Arabian Red Sea during the 2015/2016 global coral bleaching event

**DOI:** 10.1371/journal.pone.0195814

**Published:** 2018-04-19

**Authors:** Alison A. Monroe, Maren Ziegler, Anna Roik, Till Röthig, Royale S. Hardenstine, Madeleine A. Emms, Thor Jensen, Christian R. Voolstra, Michael L. Berumen

**Affiliations:** 1 Red Sea Research Center, Division of Biological and Environmental Science and Engineering, King Abdullah University of Science and Technology (KAUST), Thuwal, Saudi Arabia; 2 Marine Microbiology, GEOMAR Helmholtz Center for Ocean Research Kiel Düsternbrooker Weg 20, Kiel, Germany; 3 The Swire Institute of Marine Science, The University of Hong Kong, Hong Kong, China; Leibniz Centre for Tropical Marine Research, GERMANY

## Abstract

Coral bleaching continues to be one of the most devastating and immediate impacts of climate change on coral reef ecosystems worldwide. In 2015, a major bleaching event was declared as the “3^rd^ global coral bleaching event” by the United States National Oceanic and Atmospheric Administration, impacting a large number of reefs in every major ocean. The Red Sea was no exception, and we present herein *in situ* observations of the status of coral reefs in the central Saudi Arabian Red Sea from September 2015, following extended periods of high temperatures reaching upwards of 32.5°C in our study area. We examined eleven reefs using line-intercept transects at three different depths, including all reefs that were surveyed during a previous bleaching event in 2010. Bleaching was most prevalent on inshore reefs (55.6% ± 14.6% of live coral cover exhibited bleaching) and on shallower transects (41% ± 10.2% of live corals surveyed at 5m depth) within reefs. Similar taxonomic groups (e.g., Agariciidae) were affected in 2015 and in 2010. Most interestingly, *Acropora* and *Porites* had similar bleaching rates (~30% each) and similar relative coral cover (~7% each) across all reefs in 2015. Coral genera with the highest levels of bleaching (>60%) were also among the rarest (<1% of coral cover) in 2015. While this bodes well for the relative retention of coral cover, it may ultimately lead to decreased species richness, often considered an important component of a healthy coral reef. The resultant long-term changes in these coral reef communities remain to be seen.

## Introduction

Increasing global temperatures caused by climate change have negatively impacted coral reefs, resulting in an increase in the frequency of large-scale bleaching events [1]. Corals live in specific habitats, requiring limited ranges of salinity, nutrients, and temperature. Even small fluctuations of 1°C (for several weeks) above this range can stress corals, which then expel their intracellular symbiotic zooxanthellae causing coral bleaching [2]. The United States National Oceanic and Atmospheric Administration (NOAA) declared 2015–2016 to be a global coral bleaching event, the third in the past 20 years. It has been considered the longest and most widespread global coral bleaching event with some reefs in Hawai’i and the Great Barrier Reef experiencing severe bleaching twice [3–5]. Several areas that do not often experience high rates of bleaching during El Nino years were strongly affected including those in subtropical Hong Kong, reefs in Western Australia, and the central and southern Red Sea [6–8]. Coral bleaching has now become the main driver of coral reef degradation globally [4,9].

The Red Sea already has summer temperatures well above the average maxima of most coral reef ecosystems of the Atlantic, Indian, and Pacific Oceans [8,10,11]. Remotely-sensed sea surface temperature (SST) maxima range from an average of 31.3°C (± 1.1°C) in the southern Red Sea to 26°C (± 1°C) in the far north [7]. Additionally, the Red Sea also has remarkable differences in salinity and primary productivity along its latitudinal gradient, yet coral reef ecosystems are maintained throughout the entire gradient [12,13,14]. Despite these unique conditions little research has been conducted on the responses of Red Sea corals to thermal stress, and until recently long-term *in situ* environmental data from central Red Sea coral reefs were absent [15–17]. Cantin et al. [9] described previous thermal stress events in the central Red Sea using historical growth rates of *Diploastrea heliopora* derived from skeletal cores. Growth rates decreased in the early 1940s and in 1998, the year of the first documented global coral bleaching event [10]. A more recent measurement (2012–2013) of seasonal calcification rates of three common reef-building corals also indicates that summer temperatures currently exceed the optima of those three corals in the central Red Sea [18]. Numerous observations of coral bleaching were made in 1998 throughout the central and southern Red Sea coinciding with the decreased growth rates found by Cantin et al. [8] as well as very high SSTs (33.7°C) [7,19,20]. High levels of coral mortality followed the 1998 bleaching event from as far north as Rabigh (~23° N) to the Farasan Islands (~16° N) in the south [19].

In 2010, another bleaching event was directly observed in offshore, midshelf, and inshore reefs of the central Saudi Arabian Red Sea and was recorded to extend to depths of 15m [21]. Bleaching was most severe in the inshore reefs and at the shallowest depths (5m). Furby et al. [21] assumed the bleaching to be isolated in the central Red Sea based on observations reported by recreational divers in other areas along the Saudi Arabian coastline. Most of the sampled reefs were dominated by the families Pocilloporidae, Acroporidae, Poritidae, and the former Faviidae (most Red Sea species formerly placed within Faviidae are now in Merulinidae [22]). The most severe bleaching was found in the less abundant families Agariciidae and Fungiidae, as well as the genus *Galaxea* [21]. Notably, high levels of bleaching observed in some families (such as Acroporidae) (~35% bleached colonies), in combination with their high abundance on the reef, led to substantial community changes on some reefs [21]. Follow-up sampling 8 months later revealed a general decrease in coral cover and species richness across all reefs surveyed. The most significant community changes were found in the two inshore reefs that were surveyed; these were previously dominated by Acroporidae, but by 2011 had shifted to less than 5% of live coral and were dominated by Poritidae [21].

In this study, we present results from *in situ* observations of the central Red Sea during the 2015 global coral bleaching event. Coral bleaching was reported and informally observed on these reefs in late August 2015. In September 2015, we undertook formal *in situ* observations and analyzed the bleaching susceptibility of different taxa at different depths and distances from shore. We aimed to identify taxa highly affected by thermal stress and establish baseline data of reef composition before the occurrence of coral mortality.

## Materials and methods

### Data collection

**Fig 1:**
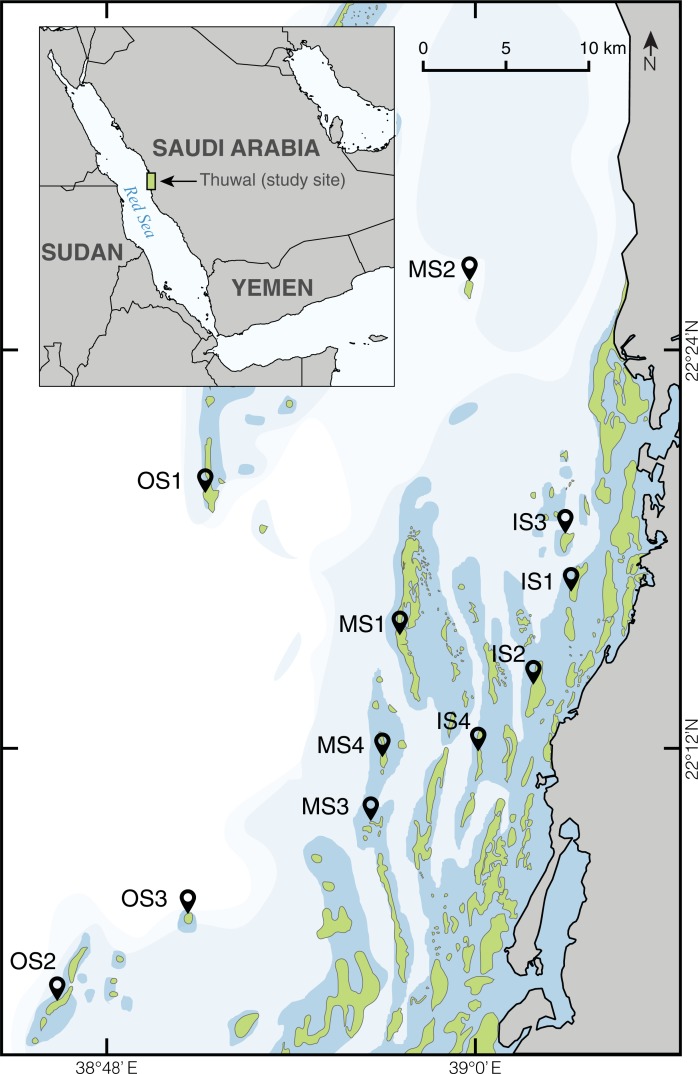
Map of the 11 reefs surveyed off the coast of Thuwal, Saudi Arabia in the central Red Sea in September 2015. Abbreviations are as follows: OS (offshore), MS (midshelf), and IS (inshore). Modified and reprinted from [26] under a CC BY license, with permission from Springer Nature, Coral Reefs (2017)(S2 File).

From September 8 to 17, 2015, eleven reefs off the coast of Thuwal, Saudi Arabia, were surveyed (Fig 1). Surveys were conducted at the same sites surveyed by Furby et al. [21] to provide a direct comparison to the 2010 bleaching event. Additional inshore and midshelf reefs were added to this study to obtain a more comprehensive survey of potentially impacted reefs. At each site, three 10m transects were assessed at each of three depths: 5m, 10m, and 15m. Benthic cover was recorded using the same method used in the surveys of the 2010 bleaching event [21] (*i*.*e*., using the line intercept method described generally by English et al. [23]) following details used by Berumen et al. 2005 [24](S1 Table). All organisms were identified to the most specific taxonomic level possible, although some grouping was necessary to enable comparisons due to low abundance in some taxa. This also alleviated potential misidentifications at the species level. Corals exhibiting loss of coloration (pale or pure white) on at least 20% of their surface were considered "bleached" for analysis purposes rather than using ‘patchy bleaching’ vs ‘fully bleached’ categories (*sensu* [25]).

### Data analyses

Bleaching was quantified as in Furby et al. [21], comparing the intercept length of bleached corals to the total intercept length of all hard corals (bleached or not) to determine a bleaching percentage (%) for each transect (or for a given taxon within a transect). Non-transformed data were used to test for differences in bleaching prevalence between 2010 and 2015 (paired *t*-test). Before running multivariate statistical analysis, bleaching percentage data were normalized using a square root transformation. Spatial patterns were identified using a two-way ANOVA with depth levels and distance from shore as fixed factors, and with bleaching percentage as the response variable. A Tukey’s post-hoc test was then run to determine the differences within the factor levels. All statistical tests were run in R 3.4.0 [27].

### Environmental data

**Fig 2:**
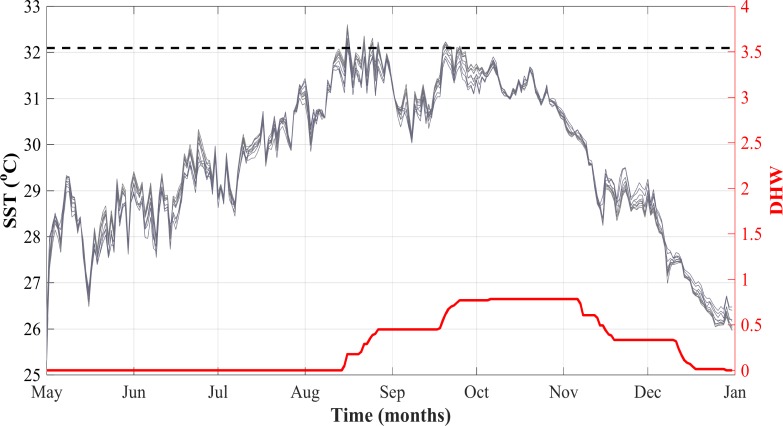
Sea surface temperature (SST), degree heating weeks (DHWs), and bleaching threshold at each reef location from May 1, 2015 to December 31, 2015. SST is represented by the gray shaded lines and the bleaching threshold temperature (averaged across all 11 reefs) is shown by the dashed black line. Both temperature values are shown in °C. The averaged DHWs across all 11 reefs are depicted by the solid red line. All data was downloaded from NOAA’s CRW 5km daily product [28].

SST and degree heating weeks (DHW) data for each reef’s coordinates from 2013–2016 were downloaded from the NOAA Coral Reef Watch (CRW) 5km daily product [28]. This data also included several other measurements including NOAA’s coral bleaching hotspot calculation which is a measurement derived by subtracting the MMM (maximum monthly mean) from the daily SST measurement [29]. The MMM is the mean climatological SST of the hottest month and is calculated based on 7 years of satellite data for each pixel in NOAAs CRW product [29]. The data output from NOAA’s CRW product provides a hotspot value and the daily SST value so we used these values to calculate the MMM for each reef, then these values were averaged to obtain a mean MMM for the study area (31.1°C) [28]. The bleaching threshold is calculated by adding 1°C to the MMM [29], so when creating Fig 2, 1°C was added to the averaged MMM for the study area to represent the bleaching threshold (32.1°C) for the study area [29].

## Results and discussion

**Fig 3:**
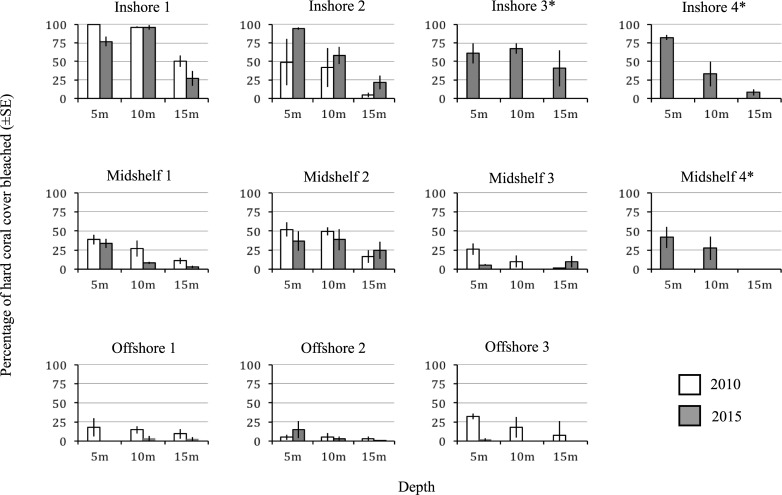
Percentages of bleached scleractinian corals in 2015 (gray bars) at 11 sites in the central Saudi Arabian Red Sea and at each of three depths compared to measurements in 2010 (white bars, from [21]). The bars represent average percent bleaching (± SE) measured on 3 replicate 10 m line-intercept transects. Sites Inshore 3, Inshore 4, and Midshelf 4 were not surveyed in 2010, denoted by an *.

Reefs in the central Saudi Arabian Red Sea were not immune to the impacts of the 2015/2016 global coral bleaching event. The reefs generally showed a similar pattern of bleaching as observed in 2010 [21]. Among the eleven sites surveyed in 2015, inshore reefs showed the highest levels of bleaching (53.7% ± 14.6%) of hard coral line-intercept length), while offshore reefs only experienced an average of 2.2% ± 2.7% bleaching (Table 1). Midshelf reefs displayed intermediate levels with only 19.2% ± 8.1% of hard coral cover bleached (Table 1). Distance from shore had the most significant impact on bleaching susceptibility (ANOVA, df = 2, F = 29.4293, p<0.0001). We then used Tukey’s post-hoc test to look at the major differences among the three tested categories (inshore, midshelf, and offshore). All categories of distance were considered significantly different from each other and inshore reefs showed the highest occurrence of bleaching. Depth also had a significant impact on bleaching presence at each reef (ANOVA, df = 2, F = 6.5335, p = 0.005). Most reefs experienced the most extensive bleaching at shallow depths (e.g., Inshore 2 had 95% ± 1.3% bleaching at a depth of 5m) (Fig 3). This is corroborated by the Tukey’s post hoc test results, as 5m was significantly different from 15m. However, neither 15m or 5m were significantly different from 10m. There was no significant difference within the depth and distance interaction (ANOVA, df = 4, F = 0.8727, p = 0.495). These results are similar to depth patterns of bleaching in reefs around the world, and in the Red Sea in 2010, in that shallow water corals and inshore reefs are typically the most susceptible to bleaching [21, 30, 31].

**Table 1:** Surveyed reefs in the central Saudi Arabian Red Sea with latitude and longitude coordinates, survey date, DHWs for the date of the survey, and percentage of corals bleached (± SE).

Site	Reef Name	Coordinates	Survey Date	DHW at time of survey	% Hard Coral Bleached	% *Porites* Bleached	% *Pocillopora* Bleached	% *Acropora* Bleached
Inshore 1	Tahla	N 22° 16.4988' E 39° 02.9804'	14-Sep-15	0.172	66.7 ± 10.9	62.2 ± 12.2	100 ± 0	87.5 ± 12.5
Inshore 2	Fsar	N 22° 14.1489' E 39° 01.8209'	13-Sep-15	0	58.2 ± 11.4	41.9 ± 11.6	100 ± 0	58.8 ± 16.9
Inshore 3	Abu Shosha	N 22° 18.2171' E 39° 02.8246'	16-Sep-15	0.6819	48.8 ± 10.8	34.5 ± 12.8	79.5 ± 12.7	54.1 ± 17.6
Inshore 4	Shaab	N 22° 12.0708' E 38° 59.9534'	13-Sep-15	0.5251	41.1 ± 12.1	35.2 ± 14.6	60 ± 24.5	77.1 ± 15.9
Midshelf 1	Al Fahal	N 22° 15.1084' E 38° 57.3863'	15-Sep-15	0.7194	15.0 ± 5.1	23 ± 12.1	3.7 ± 2.6	6.2 ± 6.2
Midshelf 2	Qita Al-Kirsh	N 22° 25.5413' E 38° 59.7357'	14-Sep-15	0.8285	33.6 ± 6.7	39.1 ± 9.1	4.7 ± 4.7	50 ± 22.4
Midshelf 3	Umm Al Kiethl	N 22° 10.1160' E 38° 56.4490'	17-Sep-15	0.3483	5.1 ± 2.6	11.8 ± 9.8	3.8 ± 3.8	0 ± 0
Midshelf 4	Umm Albalam	N 22° 11.7659' E 38° 56.9312'	8-Sep-15	0.3483	23.0 ± 8.6	19.9 ± 10.1	21.9 ± 14.5	17.5 ± 12.8
Offshore 1	Shi'b Nazar	N 22° 20.4558' E 38° 51.1270'	17-Sep-15	0.5139	2.0 ± 1.4	0.7 ± 0.7	5.6 ± 5.6	0 ± 0
Offshore 2	Abu Madafi	N 22° 04.5940' E 38° 46.5040'	16-Sep-15	0.4756	6.1 ± 4.0	10 ± 10	8 ± 4.1	0 ± 0
Offshore 3	Al-Mashpah	N 22° 06.7039' E 38° 50.5519'	16-Sep-15	0.3293	0.6 ± 0.6	0 ± 0	0 ± 0	0 ± 0

Percentages show mean proportions of cover for all hard corals and the most abundant genera that were bleached in 2015 (all depths combined). DHWs were downloaded using the reef coordinates from NOAAs CRW 5km daily product [28].

**Fig 4:**
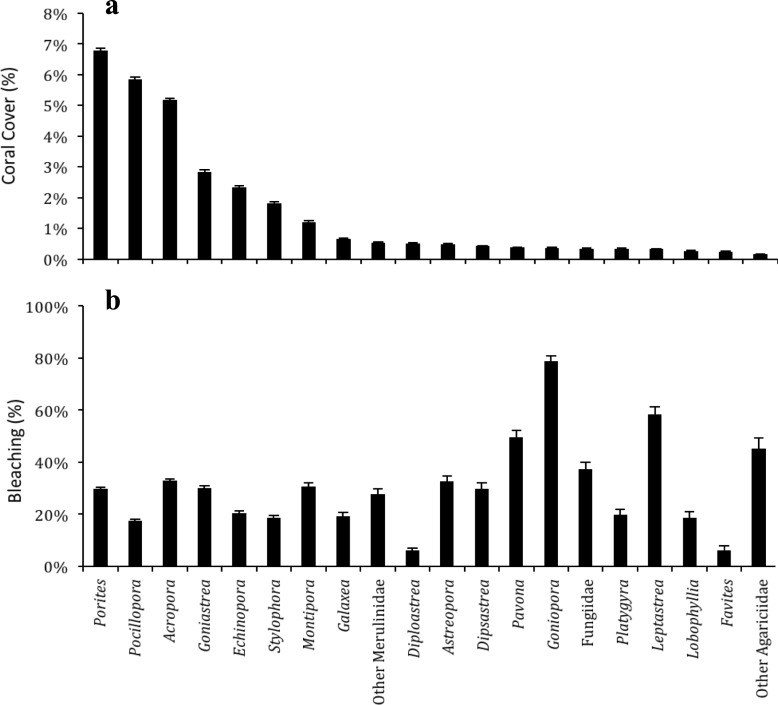
Percentage of scleractinian coral cover and their sensitivity to thermal stress during a bleaching event in the central Saudi Arabian Red Sea. **a** Average percent coral cover of the 20 most common taxa on all 10-m transects. **b** Average percent of bleached coral cover within each taxon.

Within the surveyed sites the most abundant genera included *Porites*, *Pocillopora*, and *Acropora*, each contributing to > 5% average benthic cover on the transects (Fig 4). However, compared to other coral genera, these three had comparatively low levels of bleaching (an average of < 40% of cover within each genus was bleached). (In these calculations average or relative benthic cover refers to the amount of bleaching divided by the entire benthic cover recorded, while within-taxa abundance only compares each taxa to itself.) The similarity of bleaching in the 3 abundant genera in this study is an interesting contrast to recent observations in Indonesia where *Porites* was more susceptible to bleaching than *Acropora* and other branching corals [32], but it is consistent with the situation observed in the central Red Sea bleaching event in 2010 [21]. The thermal tolerance of *Porites* could be due to the high diversity found in its *Symbiodinium* community in the Red Sea suggesting symbiont flexibility [33]. Only a few groups experienced within-taxa bleaching levels > 50%, including *Goniopora*, *Pavona*, and *Leptastrea* (Fig 4). Notably, each of these genera make up for less than 1% of the reef’s benthic cover. The Agariciidae family appeared the most affected by thermal stress, which was also the case during the 2010 and 1998 bleaching events [19, 21]. The high prevalence of bleaching in this family could account for its low abundance on all reefs in the area sampled. However, susceptibility to bleaching of these genera will not drastically change overall coral cover on the reefs, but it is expected to change the community composition and reduce species richness. The more concerning results are those of the main reef builders (*i*.*e*., *Acropora* and *Pocillopora*) where we observed a range of 50–100% within-taxa bleaching rates on inshore reefs combined with high absolute abundance (Table 1). If the bleaching resulted in mortality of the colonies, this could be detrimental to the ecological community of the inshore reefs. Only two genera (*Diploastrea* and *Favites*) showed low levels of within-taxa bleaching (6%), suggestive of resistance to thermal stress, although these were very rarely observed on our transects (Fig 4). Furby et al. [21] reported two other bleaching-resistant coral families, Astrocoeniidae and Euphyllidae, but again these were rarely observed in 2010 and previously in 2008 [34]. It is not clear whether their rarity is linked to their disturbance history.

At the time of the surveys in September 2015, the bleaching in the central Red Sea was less severe than in 2010. Offshore reefs experienced only 2.2% ± 2.7% bleaching in 2015, while in 2010 19.6% ± 4.6% of hard corals were bleached on these same reefs [21]. In the midshelf reefs this difference ranged from 41.6% ± 8.6% bleached in 2010 to 19.1% ± 8.1 bleached in 2015. However, the inshore reefs experienced similar bleaching in both events, with an average of 66.9% ± 15.5% bleaching in 2010 and 55.6% ± 14.6% in 2015 [21]. During the 2010 bleaching event, all anemones observed were bleached [35], while in 2015 bleached anemones were only observed on inshore reefs. Although there seemed to be a large difference in the percentage of bleaching, we saw no significant difference between the two years (t-test, t = 1.5488, df = 23, p-value = 0.1351).

Despite the slightly lessened bleaching severity, the temperatures of 2015 reached higher monthly maxima than 2010 (~32°C vs ~31°C), and continued for several months [36]. Neither year would have been considered in danger of severe bleaching and mortality according to the local bleaching thresholds determined by NOAA CRW [28]. Bleaching thresholds for these reefs ranged from 31.8°C to 32.7°C with the offshore reefs being at the low end and the inshore reefs being at the higher end, but averaged across all reefs to be 32.1°C (Fig 2). According to the NOAA CRW product, moderate bleaching will be seen when temperatures remain above this threshold for extended periods that equate to >4 DHWs, while severe bleaching and mortality will be seen after 8 DHWs [29]. The reefs sampled did reach above this temperature threshold for several days during this bleaching period, however it only equated to a maximum of 1.7 DHWs on midshelf 2 in late September while the other reefs remained below this DHW value (Fig 2, Table 1). The biggest discrepancy was seen at Inshore 2, which had one of the highest rates of bleaching yet experienced 0 DHW during the time of the surveys. This could be caused by several factors. For example, there may have been fine-scale differences in the *in situ* temperatures experienced by these inshore reefs in 2015 that were not captured by the remotely-sensed data used for the CRW predictions. Heron *et al*. [37] found that accumulated thermal stress measured remotely only explained 41% of observed bleaching variance, while other factors such as generic richness of the reef and specific benthic composition had an equally great effect on bleaching prevalence. This suggests that temperature stress and DHW alone are not enough to predict bleaching severity. An additional contributing factor may have been an anomalous offset between SST and temperatures at depth, potentially due to reduced inshore circulation as proposed by Furby et al. [21] as a factor in the 2010 bleaching event.

Increasing disturbances in the past two decades have been inferred and observed from several areas within the Red Sea including the 1998 global coral bleaching event that had major impacts in the central and southern regions [19,20,38]. The more recent event in 2010 caused shifts in dominant genera on several inshore reefs from branching species (*i*.*e*., *Acropora*) to massive species (*i*.*e*., *Porites*), while the midshelf reefs experienced small decreases in species richness and the offshore reefs mostly retained their original coral communities [21]. The species richness and amount of *Acropora* and *Pocillopora* colonies observed indicate the inshore reefs had begun recovery from the events of 2010; the bleaching event of 2015 could be a major setback in the recovery trajectory of these reefs. If high mortality rates occurred in 2015/2016, it would most likely be similar to 2010 with a loss of the branching, fast-growing species of the inshore reefs and a further shift to *Porites* dominance. Much remains to be learned about the thermal tolerance of Red Sea corals, particularly over large spatial scales (see [7]). However, trends in bleaching and mortality from the past three global coral bleaching events suggest a conformity to large scale bleaching patterns found in the Indo-Pacific region including the reefs of the Arabian Gulf [4,39–42]

Several reports and studies indicate that bleaching was widespread throughout the central and southern Saudi Arabian Red Sea in 2015 [7,43,44]. Osman *et al*. [7] reports that bleaching prevalence increased southward with highest bleaching rates just north of the Farasan Islands, although the northern part of the Red Sea fortunately appears to remain unaffected. It therefore appears that the Red Sea bleaching event of 2015 was more similar to that of 1998 than to that of 2010; the 2015 event impacted a much larger portion of the Red Sea while the 2010 event was apparently restricted to the central region. All studies describing the taxa affected in this region, including the present study, have identified the family Agariciidae as the most severely affected by thermal stress events [19, 21]. According to Furby *et al*. [21] the abundance of corals in this family decreased eight months after the bleaching event, suggesting high rates of mortality due to bleaching. However, the bleaching prevalence and mortality of the highly abundant branching corals (such as *Acropora*, *Pocillopora*, and *Millepora)* may be more detrimental to the health and diversity of the Red Sea reefs than a similar loss in a rarely observed genus [19–21].

The long-term impacts of the bleaching in the central Saudi Arabian Red Sea in 2015 remain to be seen. Based on the recovery inferred since 2010, it is possible that these reefs have the potential to recover, barring further thermal stress. Coral community changes due to mortality by thermal stress can have further consequences on condition or abundance of many reef-associated organisms, particularly those that depend on live corals, that may not recover as rapidly [24,45,46]. According to Graham et al. [47] it took almost 10 years post-disturbance for recruitment to reach the level necessary for rapid recovery in the Seychelles. In the Arabian Gulf, almost 15 years after the 1998 bleaching event, most reefs had yet to recover to their pre-bleaching *Acropora* dominance [42,48,49]. Fortunately, reefs in the Red Sea did not experience the same severe levels of bleaching (e.g., 90% in the Seychelles), providing hope for higher local recruitment and a more rapid recovery than a reef starting from low live coral cover. Coral reefs typically show recovery trajectories on the order of 10–25 years following disturbances that reduce coral cover, although these numbers are highly dependent on reef complexity and depth [24,47, 50–52]; despite the possibility of a rapid recovery, if bleaching events affecting inshore Red Sea reefs begin occurring every few years [9], the reefs may remain in a degraded state. As in the Arabian Gulf, the severity of bleaching events combined with naturally occurring extreme environmental conditions could slow recovery through reduced reproductive output [53] or recruitment failure [49]. Some indicators of potential resilience, such as herbivorous fish abundance, suggest that these central Red Sea reefs have an already-reduced capacity for recovery [54,55], particularly regarding heavy fishing pressure on local reef fishes [56]. Additionally, an abrupt warming in the Red Sea is ongoing since the mid 1990s and coincides with increased warming throughout the worlds’ oceans. Continuation of this pattern will likely lead to more frequent thermal stress events on coral reefs [11,12,47,57]. These increasing temperatures are causing coral reefs that were once of least concern to now exhibit clear impacts from climate change, especially in the past decade as the frequency and intensity of thermal anomalies increases [4,6,40,52,58,59]. Despite its unique environmental conditions, the Red Sea is subject to the same global stressors as other reefs worldwide. While the northern Red Sea may currently represent a sanctuary for corals against the effects of climate change [7], observations of bleaching elsewhere in the Red Sea highlight the need to further monitor impacts and recovery trends to improve regional reef management.

## Supporting information

S1 Table Benthic communities. The benthic community (±SE) at each surveyed reef site. Percentages of each category recorded were averaged over all 3 depths (5m, 10m, 15m). The category ‘Other’ is anything that didn’t fit within the other 8 categories and made up less than 1% of the community at every reef. This included giant clams, macro algae, corallimorphs, ascidians, and zooanthids.
(DOCX)

S1 File Raw data. Excel file containing the original survey data.
(XLSX)

S2 File Copyright license. File containing the copyright license for Fig 1 obtained from Springer Nature through RightsLink.
(PDF)
