# Supplementary material for: In situ observations of coral bleaching in the central Saudi Arabian Red Sea during the 2015/2016 global coral bleaching event
Source: PLoS One. 2018 Apr 19;13(4):e0195814. doi: 10.1371/journal.pone.0195814 (PMC5908266; doi:10.1371/journal.pone.0195814)
Supplement: S1 Table — The benthic community (±SE) at each surveyed reef site. Percentages of each category recorded were averaged over all 3 depths (5m, 10m, 15m). The category ‘Other’ is anything that didn’t fit within the other 8 categories and made up less than 1% of the community at every reef. This included giant clams, macro algae, corallimorphs, ascidians, and zooanthids. (DOCX) [file pone.0195814.s001.docx]

| Site | Reef name | % Hard Coral healthy | % Hard coral bleached | % Soft Coral | % Sponges | % Turf Algae | % CCA | % Rubble/ Rock | % Sand | % Other |
| --- | --- | --- | --- | --- | --- | --- | --- | --- | --- | --- |
| Inshore 1 | Tahla | 16.1 ± 0.4 | 23.6 ± 0.4 | 8.2 ± 0.3 | 1.5 ± 0.1 | 10.8 ± 0.3 | 2.4 ± 0.2 | 24.4 ± 0.5 | 10.5 ± 0.3 | 2.5 ± 0.2 |
| Inshore 2 | Inner Fsar | 11.1 ± 0.3 | 15.4 ± 0.4 | 9.3 ± 0.3 | 0.6 ± 0.1 | 9.6 ± 0.3 | 2.5 ± 0.2 | 35.8 ± 0.5 | 14.2 ± 0.4 | 1.5 ± 0.1 |
| Inshore 3 | Abu Shosha | 6.0 ± 0.3 | 8.6 ± 0.3 | 9.5 ± 0.3 | 0.3 ± 0.1 | 10.0 ± 0.3 | 3.8 ± 0.2 | 19.3 ± 0.4 | 42.1 ± 0.5 | 0.4 ± 0.1 |
| Inshore 4 | Shaab | 19.1 ± 0.4 | 14.2 ± 0.4 | 10.2 ± 0.3 | 1.2 ± 0.1 | 5.6 ± 0.2 | 0.6 ± 0.1 | 35.9 ± 0.5 | 10.9 ± 0.3 | 2.3 ± 0.2 |
| Midshelf 1 | Al Fahal | 33.3 ± 0.5 | 6.9 ± 0.3 | 35.1 ± 0.5 | 0 ± 0 | 0.5 ± 0.1 | 8.3 ± 0.3 | 10.8 ± 0.3 | 1.4 ± 0.1 | 3.7 ± 0.2 |
| Midshelf 2 | Qita Al-Kirsh | 23.9 ± 0.4 | 13.4 ± 0.4 | 13.8 ± 0.4 | 1.5 ± 0.1 | 3.2 ± 0.2 | 3.6 ± 0.2 | 31.5 ± 0.5 | 7.7 ± 0.3 | 1.4 ± 0.1 |
| Midshelf 3 | Umm Al Kiethl | 31.8 ± 0.5 | 1.0 ± 0.1 | 18.2 ± 0.4 | 1.0 ± 0.1 | 5.9 ± 0.2 | 12.0 ± 0.3 | 10.2 ± 0.3 | 17.4 ± 0.4 | 2.5 ± 0.2 |
| Midshelf 4 | Umm Albalam | 28.6 ± 0.5 | 9.3 ± 0.3 | 25.1 ± 0.5 | 0 ± 0% | 13.5 ± 0.4 | 7.6 ± 0.3 | 13.8 ± 0.4 | 0.2 ± 0.1 | 1.9 ± 0.1 |
| Offshore 1 | Shi'b Nazar | 27.2 ± 0.5 | 0.6 ± 0.1 | 21.1 ± 0.4 | 3.7 ± 0.2 | 13.5 ± 0.4 | 16.8 ± 0.4 | 12.5 ± 0.3 | 1.7 ± 0.1 | 2.9 ± 0.2 |
| Offshore 2 | Abu Madafi | 28.5 ± 0.5 | 2.6 ± 0.2 | 13.1 ± 0.4 | 1.4 ± 0.1 | 18.3 ± 0.4 | 21.1 ± 0.4 | 9.4 ± 0.3 | 0.4 ± 0.1 | 5.2 ± 0.2 |
| Offshore 3 | Al Mashpa | 36.7 ± 0.5 | 0.2 ± 0.1 | 16.7 ± 0.4 | 2.1 ± 0.2 | 11.4 ± 0.3 | 16.8 ± 0.4 | 9.1 ± 0.3 | 5.5 ± 0.2 | 1.5 ± 0.1 |
